# Supplementary material for: Yiai Fuzheng decoction inhibits triple-negative breast cancer by remodeling the immune microenvironment
Source: Front Immunol. 2025 Sep 30;16:1615631. doi: 10.3389/fimmu.2025.1615631 (PMC12518410; doi:10.3389/fimmu.2025.1615631)
Supplement: Supplementary file 2 [file DataSheet1.zip › Supplementary File 2/Treatment/C_vs_D_vs_H/06_Pathway_Analysis/Enrichment_Analysis/predicted/Enrichment_Barplot.pdf]

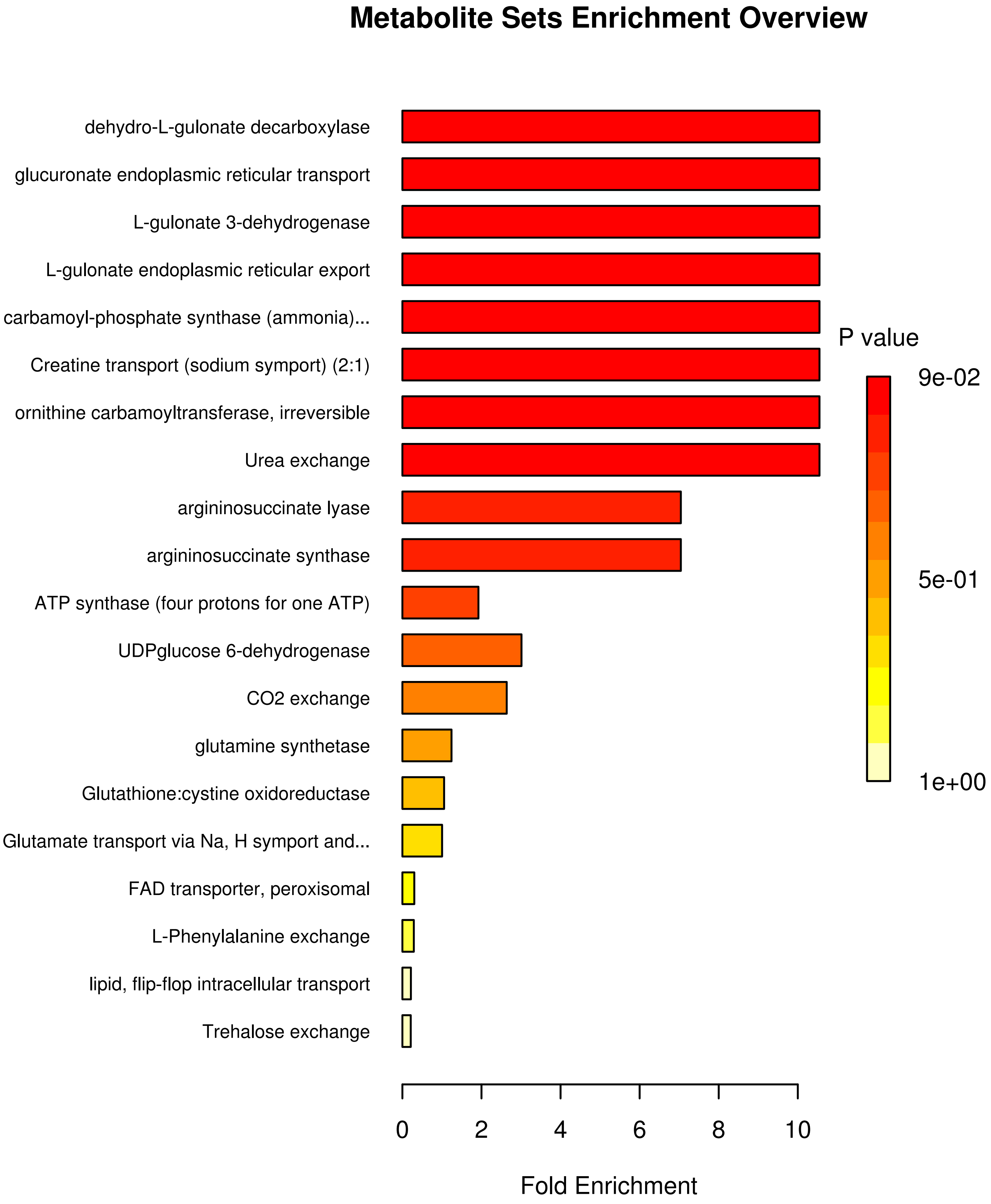
Metabolite Sets Enrichment Overview
dehydro-L-gulonate decarboxylase
glucuronate endoplasmic reticular transport
L-gulonate 3-dehydrogenase
L-gulonate endoplasmic reticular export
carbamoyl-phosphate synthase (ammonia)...
Creatine transport (sodium symport) (2:1)
ornithine carbamoyltransferase, irreversible
Urea exchange
argininosuccinate lyase
argininosuccinate synthase
ATP synthase (four protons for one ATP)
UDPglucose 6-dehydrogenase
CO2 exchange
glutamine synthetase
Glutathione:cystine oxidoreductase
Glutamate transport via Na, H symport and...
FAD transporter, peroxisomal
L-Phenylalanine exchange
lipid, flip-flop intracellular transport
Trehalose exchange
0
2
4
6
8
10
Fold Enrichment
P value
9e-02
5e-01
1e+00
